# Defect-Free Axially Stacked GaAs/GaAsP Nanowire Quantum Dots with Strong Carrier Confinement

**DOI:** 10.1021/acs.nanolett.1c01461

**Published:** 2021-06-28

**Authors:** Yunyan Zhang, Anton V. Velichko, H. Aruni Fonseka, Patrick Parkinson, James A. Gott, George Davis, Martin Aagesen, Ana M. Sanchez, David Mowbray, Huiyun Liu

**Affiliations:** † Department of Electronic and Electrical Engineering, University College London, London WC1E 7JE, United Kingdom; ‡ Department of Physics, Universität Paderborn, Warburger Straße 100, 33098 Paderborn, Germany; § Department of Physics and Astronomy, University of Sheffield, Sheffield S3 7RH, United Kingdom; ∥ Department of Physics, University of Warwick, Coventry CV4 7AL, United Kingdom; ⊥ School Department of Physics and Astronomy and the Photon Science Institute, University of Manchester, Manchester M13 9PL, United Kingdom; # Center for Quantum Devices, Niels Bohr Institute, University of Copenhagen, Universitetsparken 5, 2100 Copenhagen, Denmark

**Keywords:** nanowire, axially stacked quantum dots, defect-free crystal, carrier confinement, exciton−biexciton splitting, long-term stability

## Abstract

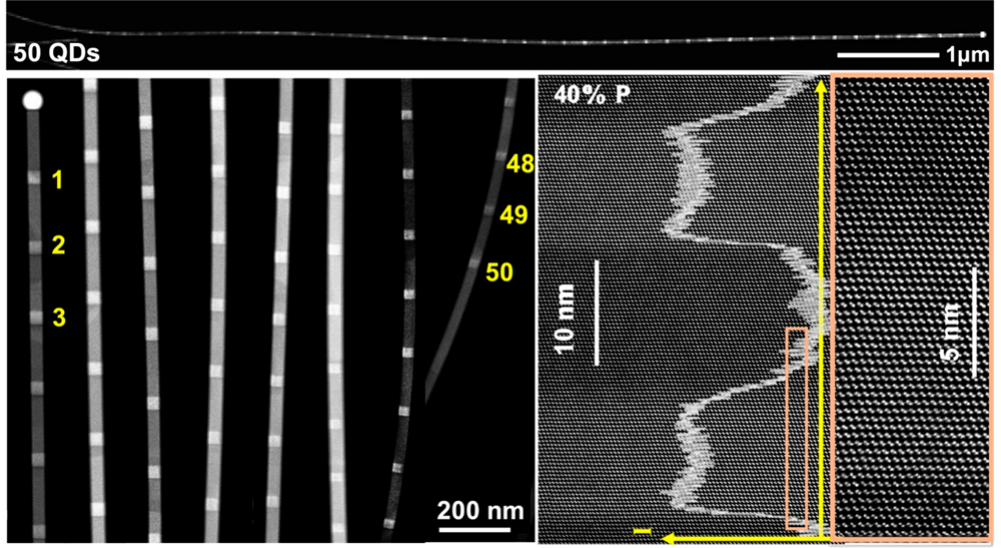

Axially stacked quantum dots (QDs) in nanowires (NWs) have important applications in nanoscale quantum devices and lasers. However, there is lack of study of defect-free growth and structure optimization using the Au-free growth mode. We report a detailed study of self-catalyzed GaAsP NWs containing defect-free axial GaAs QDs (NWQDs). Sharp interfaces (1.8–3.6 nm) allow closely stack QDs with very similar structural properties. High structural quality is maintained when up to 50 GaAs QDs are placed in a single NW. The QDs maintain an emission line width of <10 meV at 140 K (comparable to the best III–V QDs, including nitrides) after having been stored in an ambient atmosphere for over 6 months and exhibit deep carrier confinement (∼90 meV) and the largest reported exciton–biexciton splitting (∼11 meV) for non-nitride III–V NWQDs. Our study provides a solid foundation to build high-performance axially stacked NWQD devices that are compatible with CMOS technologies.

## 1 Introduction

Quantum dots (QDs) have fully quantized electronic states, permitting the fabrication of high-efficiency classical and nonclassical microelectronic and optoelectronic devices.^1,2^ For example, nanosized lasers can be achieved by vertically stacking a large number (e.g., 50) of homogeneous QDs.^3^ Enhanced complexity can be achived by closely stacking two or more QDs to achieve coupling/wave function entanglement.^4−6^ These QD molecular systems have been proposed as novel electromagnetic resonators, quantum gates for quantum computing, and thermoelectric energy harvestors.^7−10^

To date, the vast majority of QD physics and device studies have utilized self-assembled QDs, whose formation is the strain-driven Stranski–Krastanov growth mode.^11^ However, the resulting strain field makes the growth of high-quality vertically stacked identical QDs very challenging.^6^ Horizontally coupled QD pairs in thin-film structures have been studied, but the center-to-center distance between two dots is large (>30 nm), resulting in significantly weaker coupling compared with the few nanometers of separation of vertically stacked QDs.^9^ Self-assembled QDs have a number of other significant disadvantages, including formation at random positions, large inhomogeneous size distributions, limited shape and size control, and restrictions on which semiconductors can be combined in a single structure.

Semiconductor nanowires (NWs) have a unique one-dimensional morphology with many potential novel applications.^12−14^ Unlike self-assembled QDs, QD formation in an NW is not generally strain-driven, permitting a greater range of semiconductor material combinations. Direct control of both QD shape and size is possible, and the position of the QDs within the NW is fully controlled by the epitaxial growth parameters. Thus, identical QDs can be closely stacked, allowing the formation of a molecular system^15^ or laser gain regions. Additional advantages include growth along the [111]B direction, which should minimize or eliminate exciton splitting, critical for the generation of entangled photons.^16,17^ In addition, the small NW diameter provides high strain tolerance, allowing the direct integration of III–V NWs with a Si platform.^18^ There have been many reports on achieving high-performance NWQDs, with promising applications.^19−22^

NW axial heterostructures, including QDs, have been studied in a number of systems,^23,24^ e.g., InAsP-InP^25^ and AlGaAs-GaAs.^26^ Achievements include very narrow spectral line widths^26−29^ and the generation of both single photons and entangled photon pairs.^16,30,31^ The majority of previous relevant studies have used NWs fabricated via the Au-catalyzed growth method,^32,33^ possibly related to the ease of structural control, including sharp QD interfaces and crystal phases.^34−36^ However, these NWs are incompatible with Si-based electronics, as Au can be incorporated into GaAs and InAs NWs at levels on the order of 10^17^–10^18^ cm^–3^.^37,38^

More recently, the nonforeign-metal-catalyzed NW growth mode has been developed.^3^ For example, GaAsSb-based multiple axial superlattices^39^ and axially stacked InGaAs QDs^3^ have demonstrated reduced thresholds for single NW lasers. However, a majority of the reports of QD growth still exhibit high densities of stacking faults, with a mixture of zinc blende (ZB) and wurtzite (WZ) crystal structures,^40^ which has a significant impact on the electrical and optical properties.^41−44^ Stacking-fault formation is an especially serious issue for QDs grown by the widely used self-catalyzed vapor–liquid–solid growth mode,^45^ as the nanosized group-III metal catalytic droplets are highly sensitive to the growth environment.^46^ A significant challenge in growing closely stacked QDs, particularly with a large composition change between the QD and barriers, is to avoid alterations in the growth environment, which may lead to the generation of a high density of defects.^40,47^ To the best of our knowledge, there is only one report of the growth of defect-free stacked heterostructures in self-catalyzed NWs, but with a diameter in the micrometer range, it is too large to give full 3D confinement.^48^ The sensitivity of the catalytic droplets to their environment increases with reduced droplet size, due, for example, to the Gibbs–Thomson effect,^49^ which makes it challenging to grow structures that are sufficiently small to exhibit true QD behavior. Thus, there has been a lack of detailed studies of defect-free axially stacked QD structures reproducibly grown by self-catalyzed methods.

Here, we report an investigation of self-catalyzed defect-free GaAs/GaAsP single and multiple axially stacked NWQDs with deep carrier confinement. With the addition of robust in situ surface passivation, the QDs exhibit excellent optical properties.

**Figure 1:**
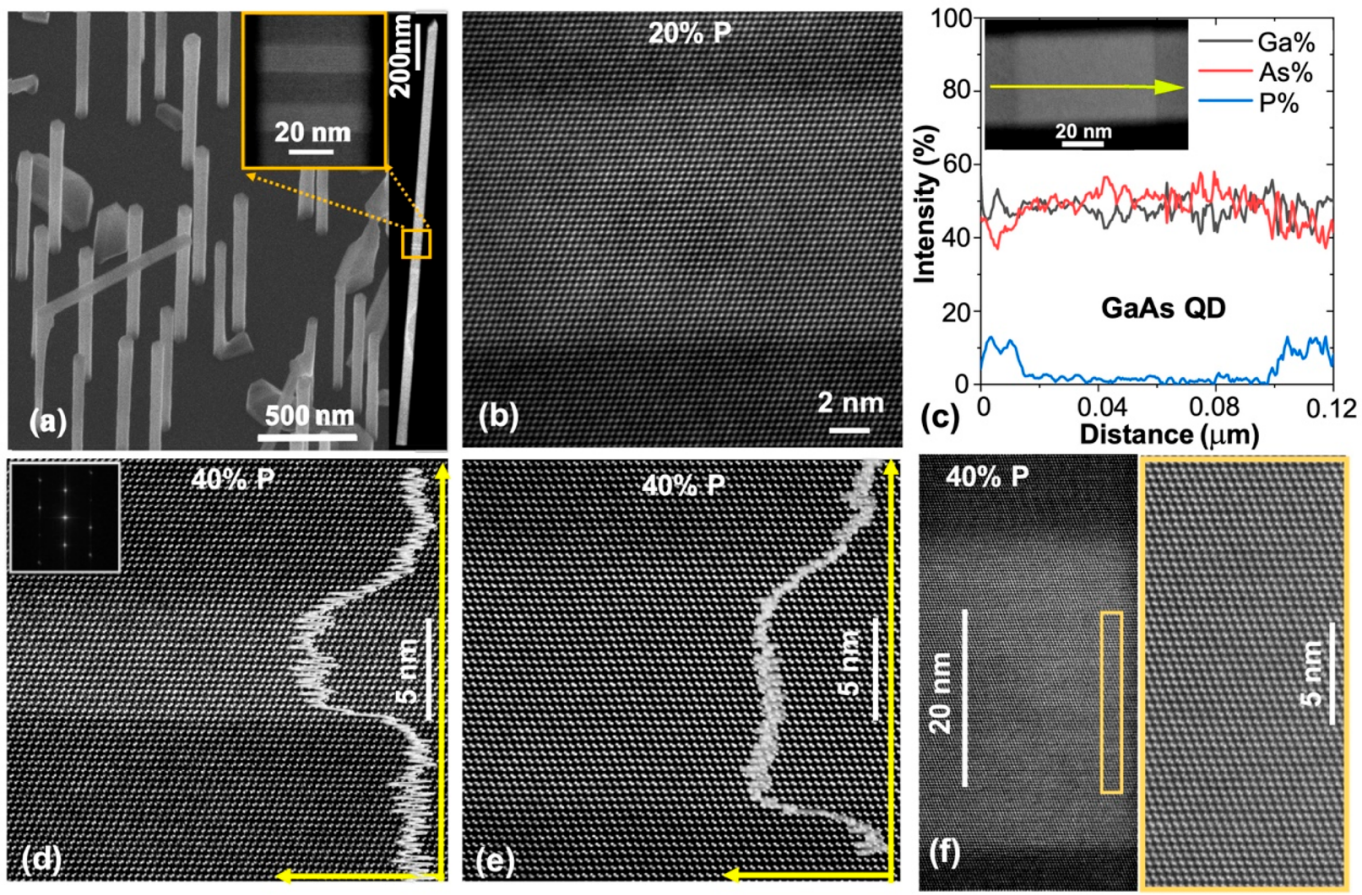
Morphology and crystalline quality of GaAsP NWs with defect-free GaAs QDs. (a) 30°-tilted SEM image of GaAs_0.6_P_0.4_ NWs with two GaAs QDs located around the midpoint of the NW (inset). (b) High-magnification ADF image of an ∼10-nm-high GaAs QD within a GaAs_0.8_P_0.2_NW. (c) EDX composition profile along the axis of the GaAs_0.8_P_0.2_/GaAs QD is shown in the inset. Annular dark-field (ADF) images of (d) ∼5-nm-high, (e) ∼10-nm-high, and (f) ∼30-nm-high GaAs QDs in GaAs_0.6_P_0.4_NWs. The overlay curves in (d) and (e) are the integrated ADF intensity profiles. The inset in (d) is the SAED pattern for the region around the QD.

GaAsP NWs with phosphorus compositions of 20 or 40% and containing GaAs QDs of varying sizes were grown using a flux compensation technique (Supporting Information S1). As shown in the scanning electron microscope (SEM) image of Figure 1a, the NWs have a highly uniform diameter of 50–60 nm along their entire length. For the single QD structure and two QD structures, the QDs are located around the NW midpoint (inset of Figure 1a). Transmission electron microscopy (TEM) analysis was performed on QDs with different sizes. Figure 1b shows an ∼10-nm-thick single GaAs QD in a GaAs_0.8_P_0.2_ NW that has a pure-ZB structure without any twins. Figure 1c shows the composition profile along the NW axis for a structure containing a taller QD. The QD is composed of almost pure GaAs. (Although EDX showed that some P was present in the QD, the amount was too low to be accurately quantified.) To provide stronger carrier confinement, QDs with higher potential barriers (GaAs_0.6_P_0.4_NWs) were grown, with heights ranging from 5 to 30 nm. All QDs exhibit a pure-ZB structure (Figure 1d–f).

The QD interface abruptness should be optimized, especially for the growth of short QDs; otherwise, the effective height of the QD is increased. The composition profiles shown in Figure 1d and e demonstrate sharp QD interfaces. The lower (GaAsP-to-GaAs) interface is only ∼6 monolayers (≡ 1.8 nm) wide, and the upper (GaAs-to-GaAsP) interface is ∼13 monolayers (≡ 3.6 nm) wide. Both interfaces are significantly more abrupt than those observed in droplet-catalyzed NWs where the group-III elements are switched (15–70 nm).^50,51^ One reason for the sharp interfaces in the present structures is the low solubility of the group-V elements within the Ga droplet at the high growth temperature of 640 °C (Supporting Information S2).^36^ The lower interface (growth of GaAs on GaAsP) is sharper than the upper one due to the As/P exchange effect (Supporting Information S3).^52^

**Figure 2:**
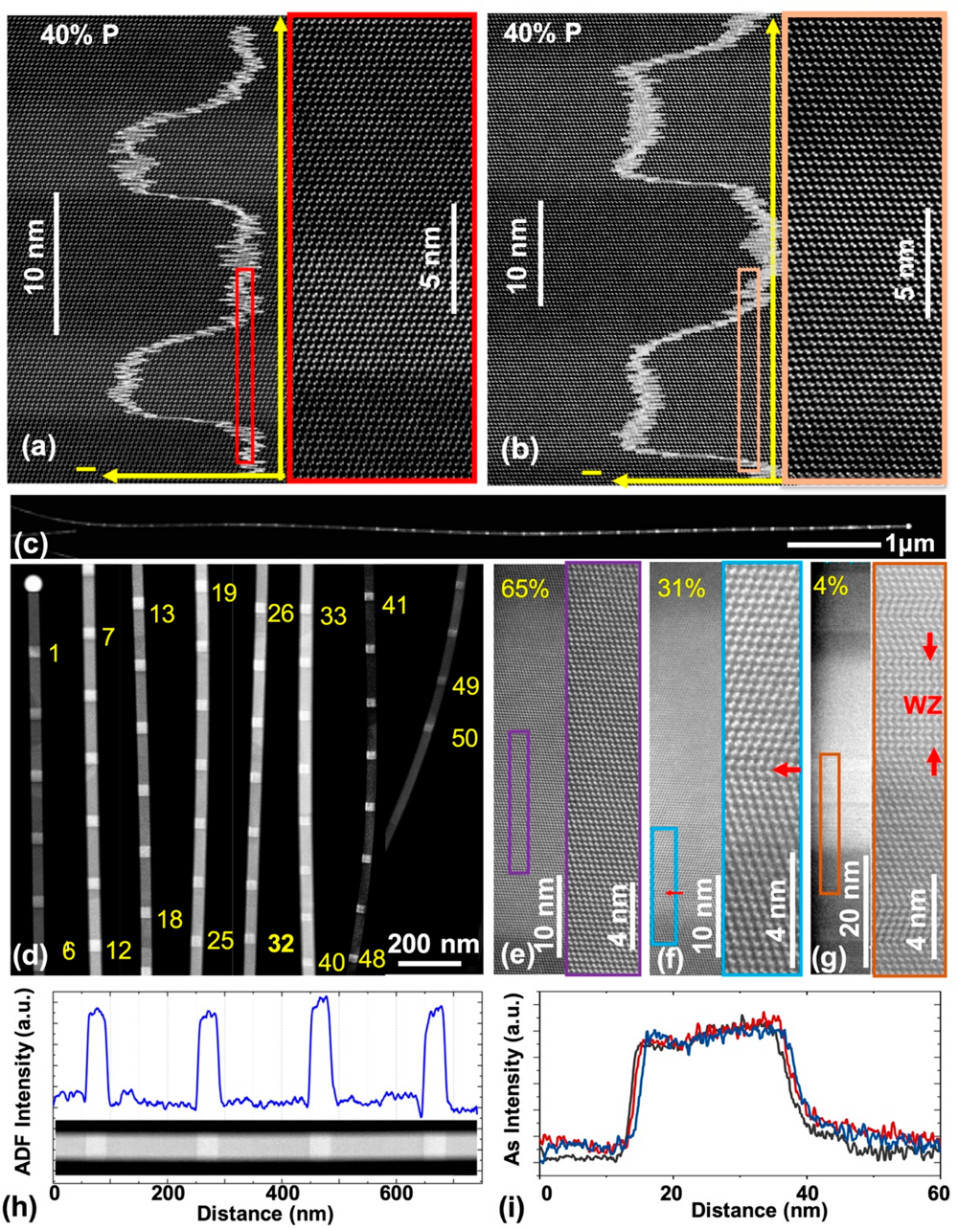
Structural properties of axially stacked GaAs_0.6_P_0.4_/GaAs QDs. (a and b) Low-magnification (left) and high-magnification (right) ADF images of 5- and 10-nm-high closely stacked pairs of QDs. The overlay curves are the integrated ADF intensity profiles. (c and d) Low-magnification ADF images of the entire NW containing 50 QDs. High-magnification ADF images of representative QDs (e) without a defect, (f) with one twin plane, and (g) containing WZ segments, as indicated by the red arrows. (h) Axial integrated intensity profiles of the NW segment shown in the inset. (i) As composition profiles for three adjacent QDs from the lower region of the NW.

To investigate if the QD separation affects their structural properties, QD pairs were grown with a small separation of ∼10 nm. As seen in Figure 2a and b, these QD pairs are stacking-fault-free with very similar composition profiles and flat interfaces, indicating that the formation of closely spaced QDs with uniform properties is possible. A sequence of 50 nominally identical GaAs QDs were grown in a GaAs_0.6_P_0.4_NW. As seem in Figure 2c and d, the NW has a highly uniform diameter of 50 nm along its length, and the round Ga droplet is retained at the tip, despite the long NW length of ∼12 μm. Both of these observations indicate a highly stable growth environment during the long growth of 1.5 h. Some (65%) of the QDs are defect-free (Figure 2e), and 31% contained a low density of twinning planes (1–4/dot, Figure 2f). Only 4% (2 dots) have very thin WZ inserts (Figure 2g). Hence, it is possible to grow a large number of axially stacked QDs with a high percentage (96%) having good structural quality. The twinning typically occurs just above the GaAsP-to-GaAs (first) interface, indicating that the observed defects are formed at the beginning of the QD growth (red arrows in Figure 2f). This suggests that a small droplet size fluctuation occurs at the start of the QD growth due to the flux switching, which can be eliminated by further optimization of the flux compensation technique. Neighboring QDs have very similar separations, heights, and composition profiles, as shown by Figure 2h and i.

**Figure 3:**
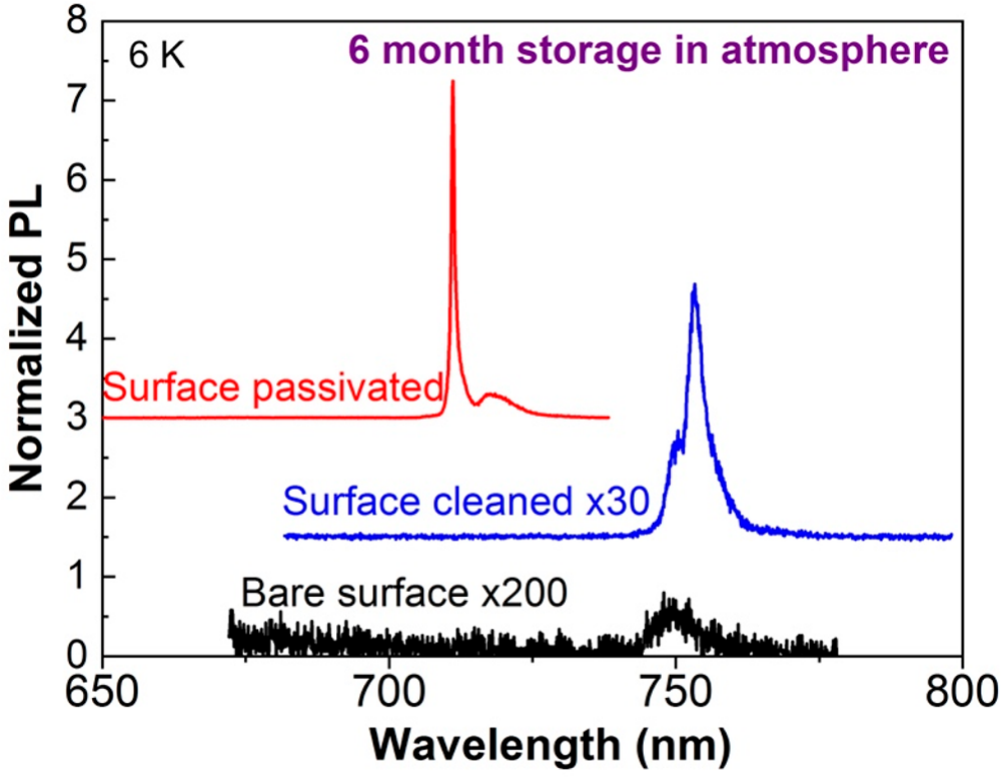
Influence of in situ passivation on the optical properties of NWQDs. μ-PL spectra of GaAs QDs in GaAsP NW with an unpassivated surface (black), without surface passivation but with surface cleaning using an ammonia solution (blue), and with surface-passivation layers (red).

The low-temperature (*T* = 6K) emission properties of the QDs were studied by performing microphotoluminescence (μ-PL) measurements on NWs that were transferred to a Si/SiO_2_ substrate, and the results are shown in Figure 3. All three NWs had been stored in an ambient atmosphere for over 6 months, resulting in the possible formation of a thin surface oxidization layer, which can result in efficient nonradiative carrier recombination.^53^ For GaAs/GaAs_0.6_P_0.4_ QDs with a bare surface (no passivation layer), only weak QD emission is observed (black spectrum). Following surface cleaning in a dilute ammonia solution (NH_4_OH:H_2_O = 1:19) for 1 min, stronger but very broad QD emission is observed (blue spectrum). This indicates that the QD quality deteriorates upon exposure to an ambient atmosphere and thus effective surface passivation is needed to achieve long-term stability. The small shift in the emission wavelength between these two spectra is in part due to inter-NW fluctuations, as it is not possible to study the same NW before and after cleaning. To improve the optical properties, ∼6 nm GaAs_0.6_P_0.4_ (to give 3D QD confinement), ∼18 nm Al_0.5_Ga_0.5_As_0.6_P_0.4_, and ∼9 nm GaAs_0.6_P_0.4_ shell layers were grown radially around the GaAsP core. These layers form a potential barrier to confine carriers within the core region and inhibit them from reaching the NW surface.^54^ These passivated NWs exhibit strong emission consisting of only a single QD peak at low laser powers, with a narrow line width of ∼500 μeV. This observation demonstrates the importance of passivation and that long-term stability of NW QDs can be achieved using only III–V materials deposited during a single growth run. This considerably simplifies the passivation and is hence suitable for mass production and large-scale applications. The QD emission for this sample is shifted with respect to the unpassivated sample as it is a significantly different structure with enhanced 3D confinement.

**Figure 4:**
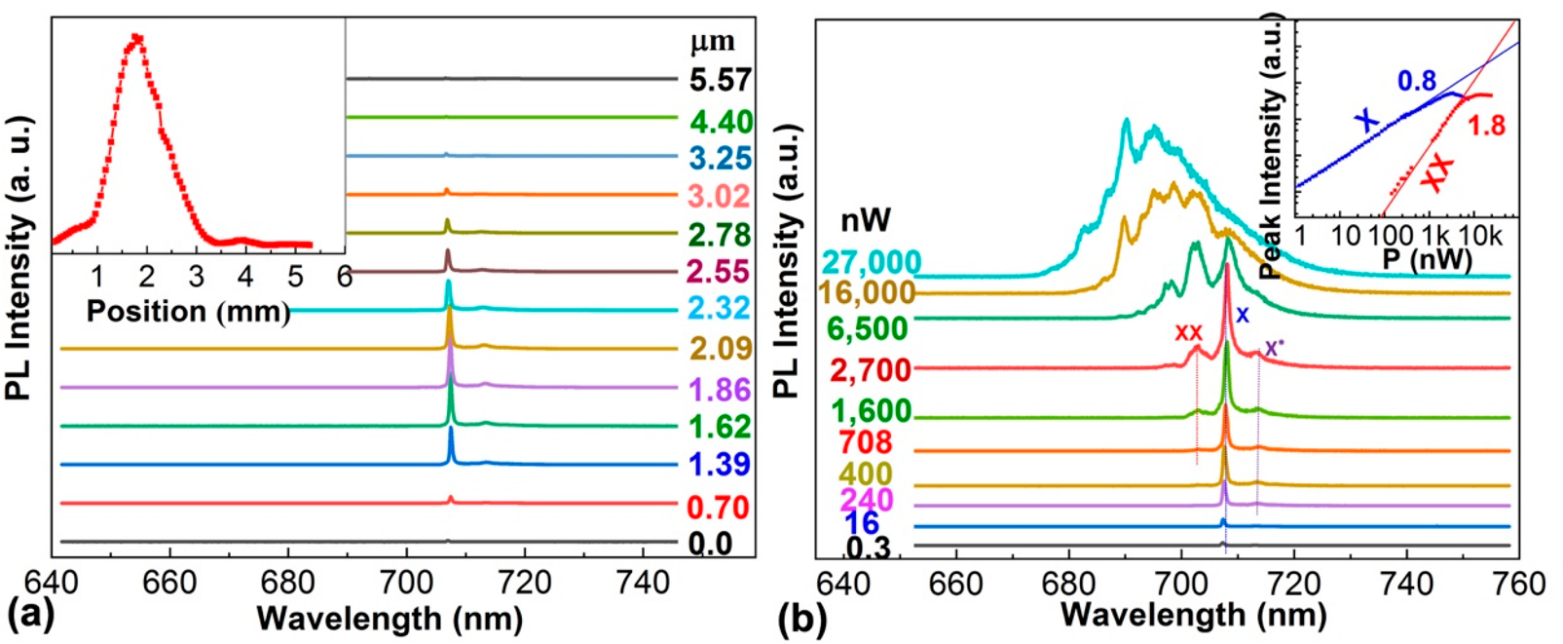
Optical properties of a surface-passivated single ∼25 nm GaAs dot in an ∼50-nm-diameter GaAs_0.6_P_0.4_NW at 6 K. (a) Position-dependent μ-PL spectra along the length of a NW. The laser power is 50 nW. The inset plots the intensity of the QD emission against the exciting laser position. (b) Power-dependent μ-PL spectra. The inset plots the intensities of two of the emission lines, X and XX, against laser power.

The detailed optical emission properties of the QDs were studied by performing μ-PL measurements on surface-passivated GaAs_0.6_P_0.4_NWs with a core diameter of 50 nm and containing a single GaAs QD of 25 nm nominal height. The QD emission at 6 K consists of a single peak at low laser powers and is highly spatially localized (Figure 4a), confirming that it originates from the GaAs QD. By fitting this localized profile (Supporting Information S4), the low-temperature carrier diffusion length is determined to be ∼0.35 μm. This indicates limited carrier diffusion at low temperatues, most likely a result of the disorderd nature of the GaAsP alloy which also influences the temperature behavior of the QD emission intensity, as discussed below.

With increasing laser power, additional emission lines appear (Figure 4b). The inset plots the intensities of the two most spectrally well-resolved lines as a function of laser power. The different gradients are consistent with exciton and biexciton recombination, and the lines demonstrate the expected high power saturation. Their separation is ∼11 meV, which is larger than previously reported values for non-nitride III–V QDs in an NW, for example, 6 meV for InAs QDs in GaAs NWs^55^ and 3 meV for GaAsP QDs in GaP NWs.^56^ Possible reasons for a large exciton–biexciton separation, which is beneficial for single photon emission at elevated temperatures, and the sign of the biexciton binding energy are discussed in Supporting Information S5. The emission spectra of Figure 4b contain a weaker feature (X*) ∼15 meV below the single exciton peak (X), most likely a singly charged (negative or positive) exciton resulting from the unequal capture of photoexcited electrons and holes by the QD (Supporting Information S6).^57^ At higher laser powers, additional features appear above the energies of the exciton and biexciton lines in the μPL spectra of Figure 4b, which are attributed to higher-order processes (Supporting Information S7).

**Figure 5:**
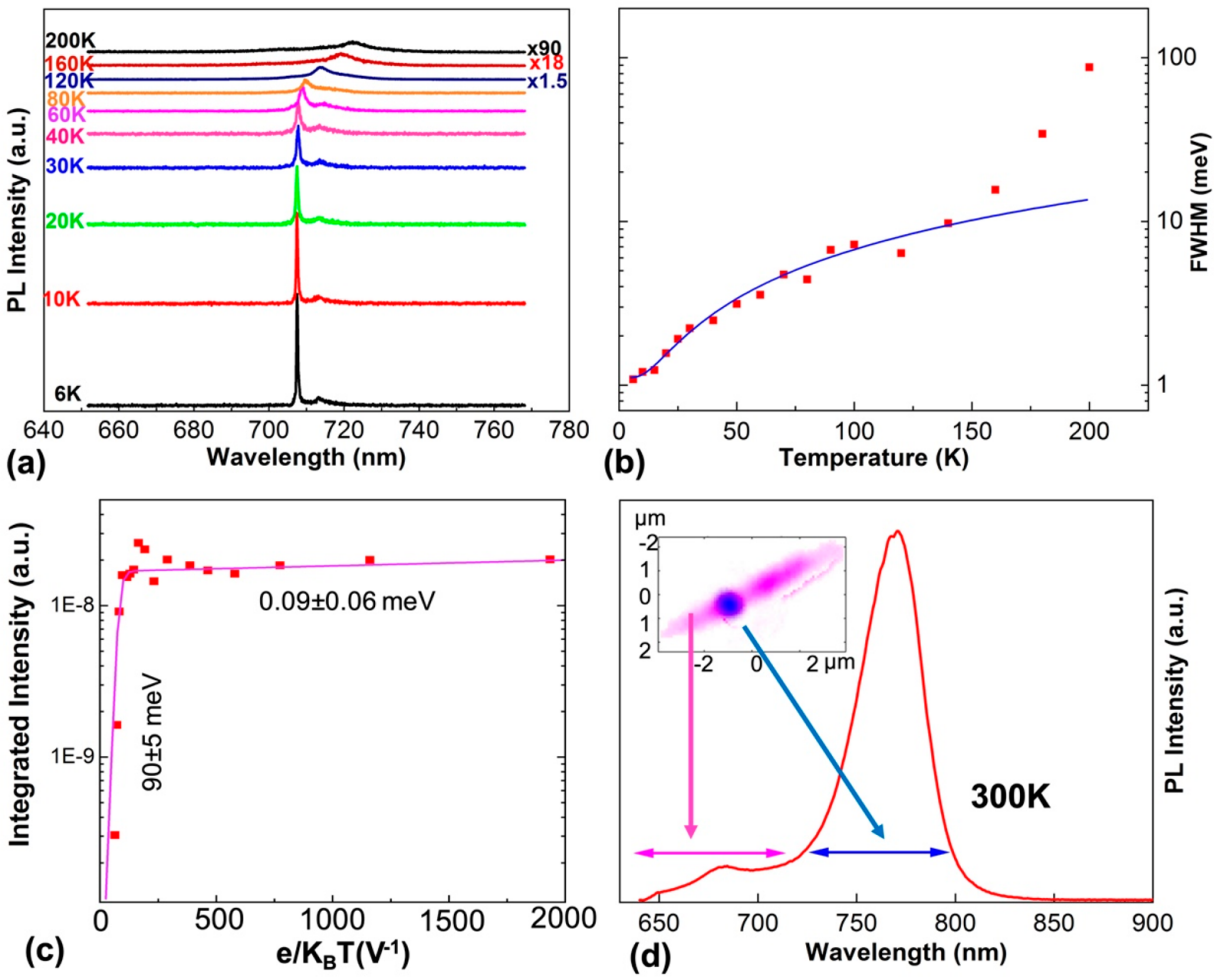
QD temperature-dependent emission properties. (a) Temperature-dependent μPL spectra of a surface-passivated GaAs_0.6_P_0.4_/GaAs QD. (b) QD emission line width and (c) integrated PL intensity plotted against temperature. The solid blue line in (b) is a fit to the low-temperature data. (d) PL spectrum at 300 K created by combining spectra recorded separately from 640 individual GaAs_0.75_P_0.25_ NWs each containing a single GaAs dot. The insets show a spectral map image of a representative NW, with two spectral bands used to create the image as indicated by the horizontal arrows in the main part of the figure.

With increasing temperature, the QD emission broadens (Figure 5a). By fitting the full width at half-maximum (fwhm) against temperature (Figure 5b) using a relevant model (blue solid line), an excited state of ∼3 meV above the ground exciton state is determined (Supporting Information S8), consistent with the relatively large size of the QD. The line width at 140 K is 9.8 meV. To the best of our knowledge, this is the first report of the emission line width at elevated temperatures for a non-nitride NWQD exhibiting long-term stability, and previous reports are limited to temperatures below ∼20 K (Supporting Information S9). A line width of 9.8 meV is comparable to the lowest published values for nitride NWQDs at elevated temperatures (above ∼100 K).^58,59^ By reducing the QD diameter and hence increasing the electron and hole confined-state separations, it should be possible to achieve smaller line widths at elevated temperatures. The large exciton and biexciton separation of 11 meV is larger than the emission line width of 9.8 meV at 140 K, allowing the exciton and biexciton lines to be spectrally resolvable, with potential application as a high-temperature single photon source.

The temperature behavior of the integrated emission intensity of a single QD exhibits complex behavior (Figure 5c). At low temperatures (≲20 K), a very small activation energy is found (∼0.1 meV). Between 30 and 70 K, the integrated intensity increases with increasing temperature, which can be explained by an increase in carrier transport in the GaAsP barrier material (Supporting Information S10). At high temperatures (≳80 K), a large activation energy of ∼90 meV is found. We have shown from studies of GaAs QWs in GaAsP NWs that both deep electron confinement and hole confinement are achieved, a result of the mixed group-V structure and the large GaAs compressive strain.^60^ Nextnano simulations indicate that the activation energy for electrons is 39% of the total energy separation of the barrier and QD band gaps, calculated as 270 meV for the present structure. This suggests an electron activation energy of 105 meV, which is in reasonable agreement with the experimentally determined value of 90 ± 5 meV.

Room-temperature emission is observable from GaAs QDs grown in GaAs_0.6_P_0.4_ NWs and also QDs in NWs with lower P compositions. Figure 5d shows a GaAs_0.75_P_0.25_/GaAs NWQD structure with only a 30-nm-thick GaAsP passivation layer. The origin of the main emission band is confirmed as arising from the QD by the spectral mapping shown in the inset of Figure 5d. High-temperature emission from QDs in a NW is typically observed for wide band gap and high exciton binding energy materials (Supporting Information S9), e.g., GaN.^61,62^ The observation of emission to relatively high temperatures further confirms the high crystalline and hence optical quality of the current QD structures.

In summary, we have demonstrated the growth of axially stacked GaAs/GaAsP NWQDs with defect-free crystal quality. The QDs have interface widths of as low as 6–13 monolayers, allowing the growth of short QDs. It is also possible to form closely separated QD pairs with highly uniform structural properties. The stacking of 50 GaAs QDs in one GaAsP NW is demonstrated, with 96% of the QDs exhibiting high crystalline quality. NWQDs degrade severely in the atmosphere, requiring the addition of (Al)GaAsP cladding layers which significantly improve the optical properties and long-term stability. Passivated QDs have a narrow line width of <10 meV at 140 K; this is the first report of the high-temperature line width for a III–V NWQD system after having been stored in an ambient atmosphere over a long period, with the value comparable to the narrowest line widths reported for nitride-based systems. The QDs exhibit a large carrier confinement energy of ∼90 meV and emission up to 300 K, consistent with a high crystalline quality, deep electron and hole confinement potentials, and effective surface passivation. A large exciton–biexciton separation (∼11 meV) is found. The narrow line width, emission at elevated temperatures, and large exciton–biexciton separation are all requirements for the high-temperature operation of quantum emitters. Values for the current structures indicate the potential of non-nitride-based NWQD quantum emitters to operate well above liquid-nitrogen temperatures, which should greatly reduce device operating costs and significantly increase the range of applications.

## 2 Materials and Methods

### 2.1 NW Growth

The self-catalyzed GaAsP NWs were grown directly on Si(111) substrates by solid-source III–V molecular beam epitaxy. If not otherwise stated, the following growth parameters were used. The core GaAs_0.6_P_0.4_ (GaAs_0.8_P_0.2_) NWs were grown with a Ga beam equivalent pressure, V/III flux ratio, P/(As + P) flux ratio, and substrate temperature of 8.41 × 10^–8^ Torr, ∼30 (40), 41% (12%), and ∼640 °C, respectively. The GaAs QDs in GaAs_0.6_P_0.4_ (GaAs_0.8_P_0.2_) NWs were grown with a Ga beam equivalent pressure and V/III flux ratio of 8.41 × 10^–8^ Torr and ∼37 (44), respectively. For samples used for optical measurement, GaAsP shells on GaAs_0.6_P_0.4_ (GaAs_0.8_P_0.2_) NWs were then grown with a Ga beam equivalent pressure, V/III flux ratio, P/(As + P) flux ratio, and substrate temperature of 8.41 × 10^–8^ Torr, 110 (86), 49% (42%), and ∼550 °C, respectively. Al_0.5_Ga_0.5_As_0.6_P_0.4_ (∼18 nm, used to block carriers from reaching the surface) and GaAs_0.6_P_0.4_ (∼9 nm, used to protect the AlGaAsP shell) shell layers were grown on the GaAs_0.6_P_0.4_ core. The Al_0.5_Ga_0.5_As_0.6_P_0.4_ shell was grown with an Al beam equivalent pressure, Ga beam equivalent pressure, V/III flux ratio, P/(As + P) flux ratio, and substrate temperature of 6.33 × 10^–8^ Torr, 8.41 × 10^–8^ Torr, 160, 49%, and ∼550 °C, respectively. The substrate temperature was measured with a pyrometer.

### 2.2 Scanning Electron Microscope (SEM)

The NW morphology was measured with a Zeiss XB 1540 FIB/SEM system.

### 2.3 Transmission Electron Microscopy (TEM)

TEM specimens were prepared by simple mechanical transfer of the nanowires from the as-grown substrate to a holey carbon grid. The TEM measurements were performed with a JEOL 2100 and doubly corrected ARM200F microscopes, both operating at 200 kV.

### 2.4 Photoluminescence (PL)

μ-PL spectra were obtained from single NWs, which had been removed from the original substrate and transferred to a new Si wafer. μPL spectra of single NWs were excited by a cw 515 nm diode laser. The samples were measured under vacuum inside a continuous flow cryostat (base temperature 6 K). The incident laser was focused with a 20× long-working-distance microscope objective to a spot size of ∼1 μm diameter. The resultant PL was collected by the same microscope objective and focused into a 0.75 m spectrometer, where the spectral components were resolved and detected using a 300 lines/mm grating and a nitrogen-cooled Si CCD. The spectral resolution was ∼0.5 meV. Higher-resolution measurements were recorded using an 1800 lines/mm grating with a resolution of 0.09 meV.

Room-temperature spectra were excited with 280 μW of 632.8 nm laser light focused to a spot size of 0.8 μm diameter. Spectra of 898 individual NWs were recorded, and the average of 640, which showed QD emission, was used to create the spectrum of Figure 5d.

## Supporting Information Available

Flux-compensation growth method; reduced reservoir effect for As and P in Ga metal droplets; asymmetrical GaAs/GaAsP hetero interfaces; fitting of carrier diffusion length; sign of the biexciton binding energy; possible charged exciton emission X*; higher-order excitonic processes; scattering of the excitons by acoustic phonons; summary of published NWQD emission line widths as a function of temperature; thermal activation of carrier transport at low temperature (PDF)
