# Supplementary material for: Defect-Free Axially Stacked GaAs/GaAsP Nanowire Quantum Dots with Strong Carrier Confinement
Source: Nano Lett. 2021 Jun 28;21(13):5722–9. doi: 10.1021/acs.nanolett.1c01461 (PMC8289304; doi:10.1021/acs.nanolett.1c01461)
Supplement: Supplementary file 1 — nl1c01461_si_001.pdf [file nl1c01461_si_001.pdf]

Supporting Information

Defect-Free Axially-Stacked GaAs/GaAsP Nanowire Quantum Dots with Strong Carrier Confinement

Yunyan Zhang,[a,f] Anton V. Velichko,[b] H. Aruni Fonseka,[c] Patrick Parkinson,[d] James A. Gott,[c] George Davis,[b] Martin Aagesen,[e] Ana M. Sanchez,[c] David Mowbray[b] and Huiyun Liu[a]*

*E-mail: yunyang.zhang.11@ucl.ac.uk

a. Department of Electronic and Electrical Engineering, University College London, London WC1E 7JE, United Kingdom;

b. Department of Physics and Astronomy, University of Sheffield, Sheffield S3 7RH, United Kingdom

c. Department of Physics, University of Warwick, Coventry CV4 7AL, United Kingdom

d. School Department of Physics and Astronomy and the Photon Science Institute, University of Manchester, M13 9PL, United Kingdom

e. Center for Quantum Devices, Niels Bohr Institute, University of Copenhagen, Universitetsparken 5, 2100 Copenhagen, Denmark

f. Department of Physics, Universität Paderborn, Warburger Straße 100, 33098, Paderborn, Germany

S1. Flux-compensation growth method

To determine the factors leading to the formation of stacking faults, a comparison was made between two types of Ga-catalyzed GaAsP NWs that are formed during the growth of a single sample. These were formed within significantly different densities, and hence separation of both the NWs and clusters formed by parasitic growth between NWs. Type-I NWs are shown in Figure S1a. They exhibit a highly uniform diameter (50~60 nm) along their entire length

(3~4 μm), except very close to their base and tip where the diameter decreases. Type-II NWs are shown in Figure S1b and exhibit a noticeable tapering, with a gradually reduced diameter from the base towards the tip, indicating a decreasing droplet size during growth. Type-I NWs commonly have a pure ZB crystal structure except close to the tip and base. In contrast, the type-II NWs typically have a high density of stacking faults along their entire length. This suggests that the reduction of the droplet size correlates with the formation of stacking faults. It is found that the stacking faults at the base and tip of the NWs are generated by an unstable droplet at the start and end of the growth, when the source flux beams are switched on and off.

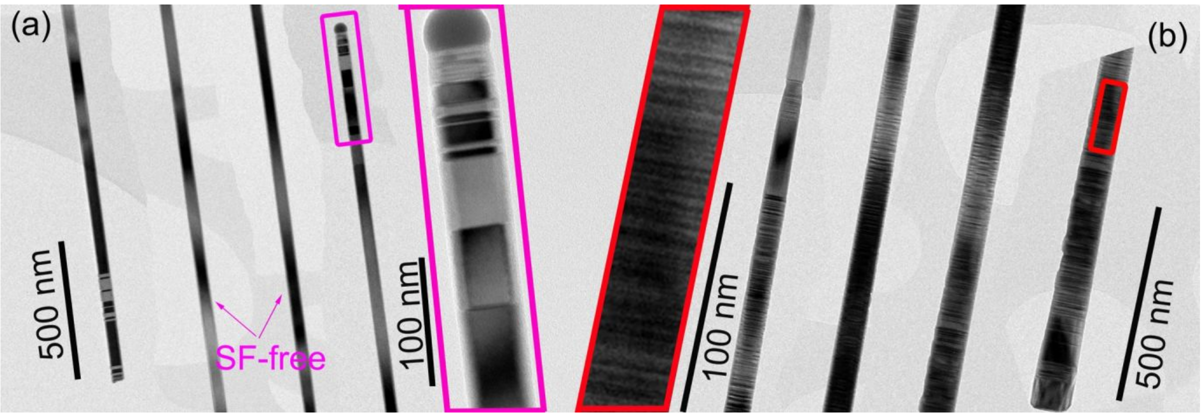

Figure S1 Relationship between droplet size fluctuation and the formation of stacking faults. Transmission electron microscopy (TEM) images of GaAsP NWs with **a)** highly uniform diameters and **b)** a gradually reducing diameter from base to tip. The dark and sharp transverse lines are stacking faults.

To grow high quality axial hetero-structures with sharp interfaces, rapid switching of the growth fluxes is required. However, these flux switches have to be performed very carefully to avoid fluctuations of the droplet size, and hence the formation of stacking faults and the potential formation of WZ segments.[1-3] To avoid these fluctuations when growing axial hetero-structures, it is crucial to maintain the droplet super-saturation, which is achieved using a "flux compensation" method. When the P flux is turned off/on, the As flux has to increase/decrease accordingly, with the change in As flux volume proportional to that of the P flux volume. Our

previous studies have shown that the P nucleation is stronger than that of As,[4] so the compensating As flux should be larger than that of the P flux change. By varying the ratio (As flux change) : (P flux change) for the growth of a number of samples, with P compositions of either 20 or 40%, we have determined the optimum value to be between 1.48 and 1.80, with the precise value depending on the NW density, inter-NW parasitic growth density, growth temperature and III-V ratio. QDs grown outside this optimum compensation range tend to have a high-density of stacking faults. Figure S2 shows TEM images obtained from GaAs QDs grown in $GaAs_{0.8}P_{0.2}$ NWs using a compensation ratio of 2.2. As this compensation ratio is outside the optimum compensation range, the QDs of different heights (10~50 nm) contain a high-density of stacking faults; in contrast to the high quality, defect-free QDs grown within the optimum compensation range. This comparison clearly demonstrates the potential of the flux compensation technique and that its use is critical in obtaining high structural quality QDs.

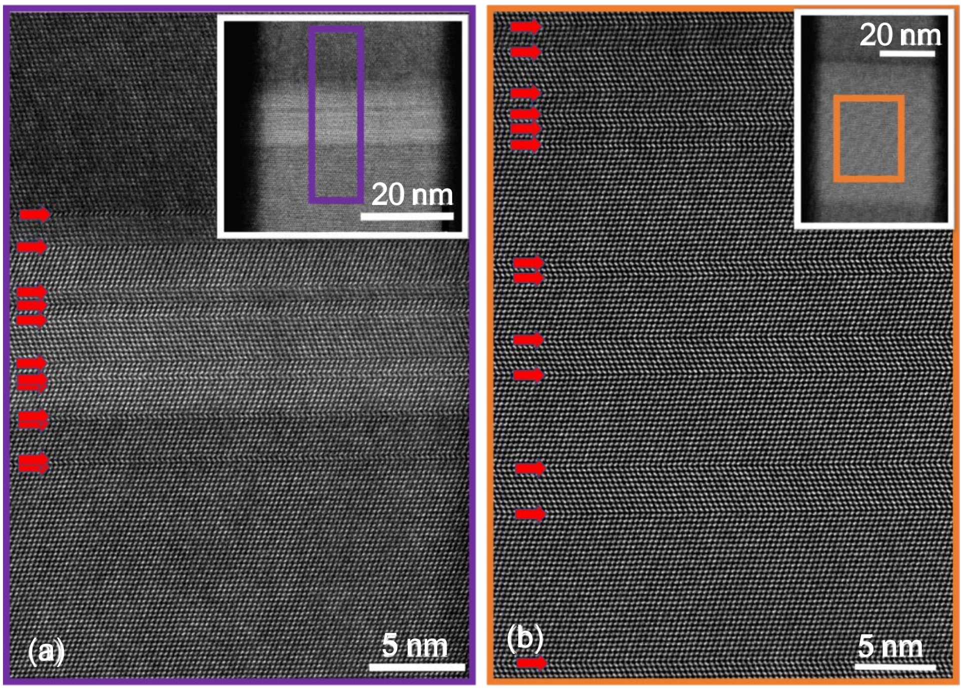

Figure S2. STEM images of GaAs QDs grown in $GaAs_{0.8}P_{0.2}$ NWs with a compensation ratio of 2.2. The QD height is ~10 nm in (**a**) and ~50 nm in (**b**). The red arrows show the locations of twins. The insets are low-magnification TEM images of the NW segments containing the QDs.

S2. Reduced reservoir effect for As and P in Ga metal droplets

The high solubility of group-III metals in the metal droplets used by the VLS method results in a significant reservoir effect; this can prevent the fabrication of sharp hetero-interfaces, especially interfaces that rely on a significant depletion of one element.[5] In contrast, group-V elements have a much lower solubility in the liquid metal compared to group-III metals, especially at high growth temperatures. This allows for very fast material depletion and switching.[6] The current GaAsP NW growth is performed at a relatively high temperature of 640 °C. This minimises the reservoir effect for As and P, resulting in the formation of sharp hetero-interfaces and the growth of almost pure GaAs QDs (Figure 1c, main paper).

S3. Asymmetrical GaAs/GaAsP hetero interfaces

During compositional switching, As/P inter-diffusion occurs, reducing the sharpness of the interface. P atoms are more strongly bonded to Ga atoms, hence it is more difficult to replace P atoms with As atoms.[7] As a result, inter-diffusion at the GaAsP-to-GaAs interface is weaker than at the GaAs-to-GaAsP interface, leading to the former interface being sharper.

S4. Fitting of carrier diffusion length

The best fit to the measured profile is obtained by convolving a 1 μm width Gaussian function, representing the laser spot size, with a second Gaussian of width 0.7 μm. As this value (0.7 μm) is significantly larger than the physical size of the QD it represents the ability of photoexcited carriers to diffuse along the NW axis, followed by their capturing into the QD. Hence, the low temperature carrier diffusion length is ~0.35 μm.

S5. Sign of the biexciton binding energy

The biexciton emission occurs at a higher energy than that of the single exciton (Figure 4 of

the main paper) indicating a negative biexciton binding energy. Although a negative binding energy is not possible in higher dimensionality structures, the complete confinement provided by a QD allows for the possibility of both types of biexciton binding energy (positive or negative). Previous reports of QDs in NWs have demonstrated both positive (e.g. In(Ga)As QDs in GaAs NWs[8]) and negative (e.g. InAsP QDs in InP NWs[9]) biexciton binding energies. The biexciton binding energy has been studied as a function of QD size in site-controlled pyramidal GaInAs/GaAs quantum dots[10] and self-assembled InAs QDs[11] . The sign of the biexciton binding energy will depend on the relative sizes of the Coulomb interactions between similar carriers (electron-electron and hole-hole) and different carriers (electron-hole). If the former dominates the latter, a negative binding energy will result. A mechanism via which this can occur is a significant physical separation between the electron and hole wave functions which enhances the Coulomb interaction between like carriers and reduces the interaction between different type carriers. In the site-controlled pyramidal GaInAs/GaAs QDs studied in Ref 10, a negative biexciton binding energy was observed for larger QDs and attributed to the effect of piezoelectric fields which separate the electrons and holes. Because of the [111] growth direction of the NWs and the strain applied to the GaAs QDs by the GaAsP barriers PZ fields are expected to be present in the current structures.

Figure S3 shows band edge profiles and the lowest electron and hole probability densities calculated using nextnano software and plotted along the central axis of the NW. The potential difference across the 25-nm high QD is ~80 meV, this gives an average PZ field of 3.2×10^6 Vm^{-1}. The combined effect of the strain modification to the band edge profiles and the strain-induced PZ field spatially separates the electron and hole wavefunctions along the NW axis, the maxima of the probability densities are separated by 9 nm. This separation is hence consistent with the observed negative biexciton binding energy observed in the current structure.

A significant physical separation of the electron and hole wave functions can enhance the biexciton binding energy; an additional contribution may arise due to the large confinement provided by the 40% composition GaAsP barriers. For example, it has been reported that InAs/AlAs QDs have a much larger exciton-biexciton splitting than InAs/GaAs QDs due to the deeper carrier confinement provided by the AlAs barriers.[12]

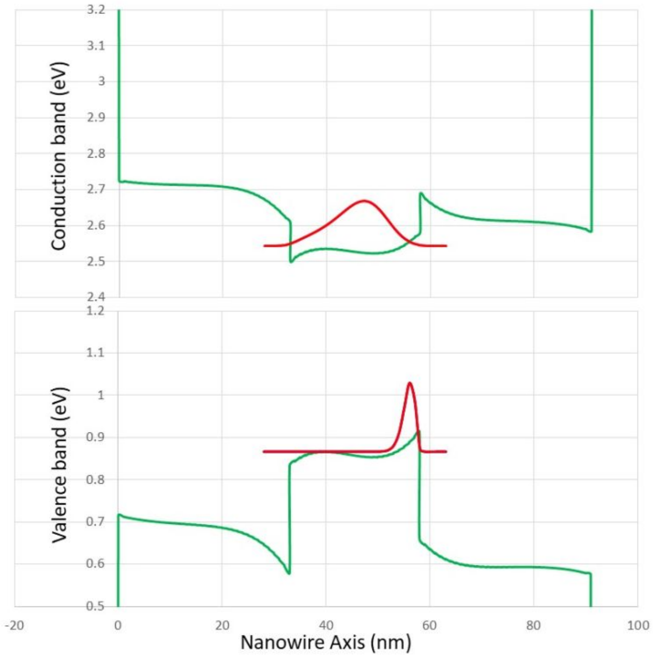

Figure S3 Calculated conduction and valence band edge profiles along the central axis of the NW showing the effects of strain and strain-induced PZ fields. The red lines show the calculated probability densities for the lowest energy QD electron and hole states.

S6. Possible charged exciton emission X*

The emission spectra of Figure 4 contain a weaker feature (X*) ~15 meV below the single exciton peak (X). A similar feature is seen in the emission of the passivated sample in Figure 3 and in other NWs from the same growth run as the NW studied in detail in the present paper. X* exhibits the same intensity behaviour with laser position as the exciton emission (inset to Figure 4a), consistent with QD related emission. The power dependence of X* is very similar

to that of the single exciton, with a linear behaviour at low powers (exponent of~0.95±0.05), followed by saturation and then intensity reduction at higher laser powers. Saturation occurs at approximately half the incident laser power observed for the saturation of the single exciton. In comparison to the intensity of the single exciton, the intensity of X* initially increases as the temperature is raised from 6 to 40 K followed by a decrease at higher temperatures. The observed behaviour of X*, particularly its intensity versus laser position and excitation power dependence, strongly suggests single exciton QD-related emission, most likely a singly charged (negative or positive) exciton resulting from unequal capture of photoexcited electrons and holes by the QD.[13] In self-assembled QDs the negatively charged exciton (X^-) is typically observed below the energy of X, with the positively charged exciton (X^+) to higher energy.[13-16] However, calculations indicated that the ordering of X, X^- and X^+ can depend on the QD structure.[17] As the current QDs have a very different shape and size compared to self-assembled dots, it is not possible to conclusively identify the nature of X*. One method to distinguish charged and uncharged excitons is photoluminescence excitation (PLE),[13] where direct excitation into the QD should create only uncharged excitons. Such measurements will form part of future studies. These may also allow the nature of the higher energy features (excited states or higher order exciton complexes), as observed in Figure 4b (and discussed in S7) at high excitation powers, to be determined.

S7. Higher order excitonic processes

At higher laser powers, additional features appear above the energies of the exciton and biexciton lines in the µPL spectra of Figure 4b. These are attributed to higher order processes, either carriers in the ground state of the QD recombining in the presence of carriers in excited states, or direct recombination from the excited states. As the separations between confined QD states is comparable with the binding energies of exciton complexes, it is not possible to

distinguish between these two mechanisms based on the current experimental data. Because of their strong spectral overlap, it is not possible to extract reliable power dependencies for these lines although over a limited power range the line at 689 nm demonstrates an exponent of 4.4, consistent with a higher order excitonic process.

S7. Scattering of the excitons by acoustic phonons

The full width at half maximum (FWHM) against temperature in Figure 5b is fitted by the solid blue line using the function:

$$\Gamma(T) = \Gamma_0 + \frac{\Gamma_a}{\exp(E_a / kT) - 1}$$

where Γ_0 is the linewidth at low temperatures (1 meV for the current QD) and Γ_a and E_a are fitting parameters. This function describes broadening via the scattering of the excitons to a higher energy state by acoustic phonons, with E_a being the energy separation of the two states.[18] The function describes the experimental data well for temperatures up to ~140K (solid blue line in Figure 5b) and gives a value for E_a of ~3 meV. Simulations performed using nextnano software[19,20] give confined electron and hole state separations for a 25 nm high and 40 nm diameter QD of ~11 and 6 meV, respectively. Hence, the determined value for E_a is consistent with exciton scattering into an excited QD state.

S8. Summary of published NWQD emission linewidths as a function of temperature

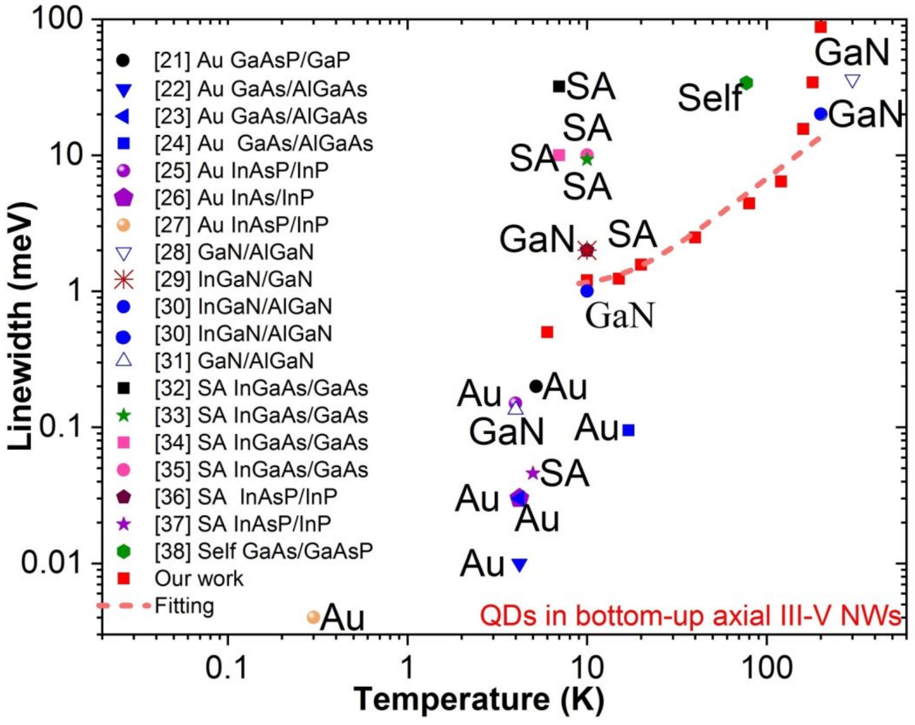

Figure S4. Emission linewidth summary for axial III-V QDs grown by bottom-up methods. The pink dashed line is the fit to our temperature dependent data (Figure 5b). "SA" indicates selective-area growth. "Au" indicates Au catalysed growth and "GaN" indicates gallium nitride-based NWs.

Table S1. Emission linewidth summary for various III-V NWQD systems

1st Author	NW type	Material system	T (K)	Linewidth (meV)
**Bottom-up III-V QDs**				
Borgstrom [21]	Au	GaAsP/GaP	5.2	0.2
Cirlin [22]	Au	GaAs/AlGaAs	4.2	0.01
Leandro [23]	Au	GaAs/AlGaAs	4.2	0.03
Heinrich [24]	Au	GaAs/AlGaAs	17.0	0.095
Haffouz [25]	Au	InAsP/InP	4.0	0.15
Dalacu [26]	Au	InAs/InP	4.2	0.03
Reimer [27]	Au	InAsP/InP	0.3	0.004
Holmes [28]	Nitride	GaN/AlGaN	300.0	36.0
Deshpande [29]	Nitride	InGaN/GaN	10.0	2.0
Deshpande [30]	Nitride	InGaN/AlGaN	10 & 200	1-2 & 15-20
Holmes [31]	Nitride	GaN/AlGaN	4.0	0.135
Tatebayashi [32]	SA	InGaAs/GaAs	7.0	32.0
Tatebayashi [33]	SA	InGaAs/GaAs	10.0	9.3
Tatebayashi [34]	SA	InGaAs/GaAs	7.0	10.0

Tatebayashi [35]	SA	InGaAs/GaAs	10.0	10
Yanase [36]	SA	InAsP/InP	10.0	2.0
Dorenbos [37]	SA	InAsP/InP	5.0	0.046
Wu [38]	Self-catalyzed	GaAs/GaAsP	77.0	34
**Our work**	**Self-catalyzed**	**GaAs/GaAsP**	**6.0**	**0.5**
**Our work**	**Self-catalyzed**	**GaAs/GaAsP**	**140.0**	**9.8**
**Other QDs in III-V NWs**				
Bavinck [39]	Crysal Phase	InP	4.2	0.023
Kremer [40]	top-down	InGaAs/GaAs	4.5	0.045
Munsch [41]	top-down	InAs/GaAs	4.2	0.004
Francaviglia [42]	radial	GaAs/AlGaAs	12.0	0.74
Heiss [43]	radial	GaAs/AlGaAs	4.2	0.029
Shang [44]	radial	GaAs/AlAs	4.2	0.037
Weib [45]	radial	GaAs/AlGaAs	5.0	1.0
Yan [46]	radial	GaAs/InP	5.5	1.28
Yu [47]	radial	GaAs/AlGaAs	4.2	0.1

Figure S4 and Table S1 summarize previous reports of emission linewidths for different III-V NWQD systems. The current work represents the first report of narrow emission linewidths for non-nitride based NWQDs above 20K. High-temperature emission from QDs in a NW is typically observed for systems with a wide bandgap and large exciton binding energy, e.g. GaN. Despite a much smaller band gap and exciton binding energy, we observe emission at 140K with a linewidth of 9.8 meV. This value is comparable with the best-reported values for nitride NWQDs.

S9. Thermal-activation of carrier transport at low temperature

At low temperatures, carriers are relatively immobile due to localisation caused by alloy fluctuations. As the temperature increases, these carriers are thermally activated from the localisation centres and so a greater number are able to diffuse and be captured by the QD. Hence, there is a region where the QD emission intensity increases with increasing temperature.

References:

(1) Priante, G.; Patriarche, G.; Oehler, F.; Glas, F.; Harmand, J. C. Abrupt GaP/GaAs interfaces in self-catalyzed nanowires. *Nano letters*, **2015**, *15*, 6036. https://doi.org/10.1021/acs.nanolett.5b02224

(2) Verheijen, M. A.; Immink, G.; de Smet, T.; Borgström, M. T.; Bakkers, E. P. Growth kinetics of heterostructured GaP− GaAs nanowires. *Journal of the American Chemical Society*, **2006**, *128,* 1353-1359. https://doi.org/10.1021/ja057157h

(3) Todorovic, J.; Kauko, H.; Ahtapodov, L.; Moses, A. F.; Olk, P.; Dheeraj, D. L. Van Helvoort, A. T. J. The effects of Sb concentration variation on the optical properties of GaAsSb/GaAs heterostructured nanowires. *Semiconductor science and technology*, **2013**, *28*, 115004. https://doi.org/10.1088/0268-1242/28/11/115004

(4) Zhang, Y.; Aagesen, M.; Holm, J. V.; Jørgensen, H. I.; Wu, J.; Liu, H. Self-catalyzed GaAsP nanowires grown on silicon substrates by solid-source molecular beam epitaxy. *Nano letters*, **2013**, *13*, 3897. https://doi.org/10.1021/nl401981u

(5) Li, N.; Tan, T. Y.; Gösele, U. Transition region width of nanowire hetero-and pn-junctions grown using vapor–liquid–solid processes. *Applied Physics A*, **2008**, *90*, 591. https://doi.org/10.1007/s00339-007-4376-z

(6) Björk, M. T.; Ohlsson, B. J.; Sass, T.; Persson, A. I.; Thelander, C.; Magnusson, M. H. Samuelson, L. One-dimensional steeplechase for electrons realized. *Nano Letters*, **2002**, *2*, 87. https://doi.org/10.1021/nl010099n

(7) Decobert, J.; Patriarche, G. Transmission electron microscopy study of the InP/InGaAs and InGaAs/InP heterointerfaces grown by metalorganic vapor-phase epitaxy. *Journal of Applied Physics*, **2002**, *92*, 5749. https://doi.org/10.1063/1.1513891

(8) Tatebayashi, J.; Ota, Y.; Ishida, S.; Nishioka, M.; Iwamoto, S.; Arakawa, Y. Optical properties of site-controlled InGaAs quantum dots embedded in GaAs nanowires by selective metalorganic chemical vapor deposition. *Japanese Journal of Applied Physics*, **2012**, *51*, 11PE13. https://doi.org/10.1143/JJAP.51.11PE13

(9) Dalacu, D.; Mnaymneh, K.; Lapointe, J.; Wu, X.; Poole, P. J.; Bulgarini, G. Reimer, M. E. Ultraclean emission from InAsP quantum dots in defect-free wurtzite InP nanowires. *Nano letters*, **2012**, *12*, 5919. https://doi.org/10.1021/nl303327h

(10) Jarlov, C.; Gallo, P.; Calic, M.; Dwir, B.; Rudra, A.; Kapon, E. Bound and anti-bound biexciton in site-controlled pyramidal GaInAs/GaAs quantum dots. *Applied Physics Letters*, **2012**, *101*, 191101. https://doi.org/10.1063/1.4765646

(11) Dal Savio, C.; Pierz, K.; Ade, G.; Danzebrink, H. U.; Göbel, E. O.; Hangleiter, A. Optical study of single InAs on In 0.12 Ga 0.88 As self-assembled quantum dots: biexciton binding energy dependence on the dots size. *Applied Physics B*, **2006**, *84*, 317. https://doi.org/10.1007/s00340-006-2327-6

(12) Sarkar, D.; Van Der Meulen, H. P.; Calleja, J. M.; Becker, J. M.; Haug, R. J.; Pierz, K. Exciton fine structure and biexciton binding energy in single self-assembled InAs⁄AlAs quantum dots. *Journal of applied physics*, **2006**, *100*, 023109. https://doi.org/10.1063/1.2209089

(13) Finley, J.J.; Ashmore, A.D.; Lemaître, A.; Mowbray, D.J.; Skolnick, M.S.; Itskevich, I.E.; Maksym, P.A.; Hopkinson, M. and Krauss, T.F. Charged and neutral exciton complexes in individual self-assembled In (Ga) As quantum dots. *Physical Review B*, **2001**, *63*, 073307. https://doi.org/10.1103/PhysRevB.63.073307

(14) Moskalenko, E.S.; Karlsson, K.F.; Holtz, P.O.; Monemar, B.; Schoenfeld, W.V.; Garcia, J.M. and Petroff, P.M. Formation of the charged exciton complexes in self-assembled InAs single quantum dots. *Journal of applied physics*, **2002**, *92*, 6787. https://doi.org/10.1063/1.1516871

(15) Cade, N.I.; Gotoh, H.; Kamada, H.; Tawara, T.; Sogawa, T.; Nakano, H. and Okamoto, H. Charged exciton emission at 1.3 μ m from single InAs quantum dots grown by metalorganic chemical vapor deposition. *Applied Physics Letters*, **2005**, *87*, 172101. https://doi.org/10.1063/1.2093927

(16) Canet-Ferrer, J.; Munoz-Matutano, G.; Herranz, J.; Rivas, D.; Alén, B.; Gonzalez, Y.; Fuster, D.; Gonzalez, L. and Martínez-Pastor, J. Exciton and multiexciton optical properties of single InAs/GaAs site-controlled quantum dots. *Applied Physics*

Letters, **2013**, *103*, 183112.
https://doi.org/10.1063/1.4828352

(17) Zunger, A. and Bester, G. Theory of excitons, charged excitons, exciton fine-structure and entangled excitons in self-assembled semiconductor quantum dots. *Physica E: Low-dimensional Systems and Nanostructures*, **2004**, *21*, 204.
https://doi.org/10.1016/j.physe.2003.11.156

(18) Gammon, D.; Snow, E. S.; Shanabrook, B. V.; Katzer, D. S.; Park, D. Homogeneous Linewidths in the Optical Spectrum of a Single Gallium Arsenide Quantum Dot. *Science*, **1996**, *273*, 87.
https://doi.org/10.1126/science.273.5271.87

(19) Fonseka, H.A.; Velichko, A.V.; Zhang, Y.; Gott, J.A.; Davis, G.D.; Beanland, R.; Liu, H.; Mowbray, D.J. and Sanchez, A.M. Self-formed Quantum Wires and Dots in GaAsP-GaAsP Core-Shell Nanowires. *Nano letters*, **2019**, *19*, 4158-4165
https://doi.org/10.1021/acs.nanolett.9b01673

(20) Birner, S.; Zibold, T.; Andlauer, T.; Kubis, T.; Sabathil, M.; Trellakis, A.; Vogl, P. Nextnano: general purpose 3-D simulations. *IEEE Transactions on Electron Devices*, **2007**, *54*, 2137-2142.
https://doi.org/10.1109/TED.2007.902871

(21) Borgström, M. T.; Zwiller, V.; Müller, E.; Imamoglu, A. Optically bright quantum dots in single nanowires. *Nano letters*, **2005**, *5*, 1439.
https://doi.org/10.1021/nl050802y

(22) Cirlin, G. E.; Reznik, R. R.; Shtrom, I. V.; Khrebtov, A. I.; Samsonenko, Y. B.; Kukushkin, S. A. Leonardo, L. Hybrid GaAs/AlGaAs Nanowire—Quantum dot System for Single Photon Sources. *Semiconductors*, **2018**, *52*, 462.
https://doi.org/10.1134/S1063782618040103

(23) Leandro, L.; Gunnarsson, C. P.; Reznik, R.; Jöns, K. D.; Shtrom, I.; Khrebtov, A. Akopian, N. Nanowire quantum dots tuned to atomic resonances. *Nano letters*, **2018**, *18*, 7217.
https://doi.org/10.1021/acs.nanolett.8b03363

(24) Heinrich, J.; Huggenberger, A.; Heindel, T.; Reitzenstein, S.; Höfling, S.; Worschech, L.; Forchel, A. Single photon emission from positioned GaAs/AlGaAs photonic nanowires. *Applied Physics Letters*, **2010**, *96*, 211117.
https://doi.org/10.1063/1.3440967

(25) Haffouz, S.; Zeuner, K. D.; Dalacu, D.; Poole, P. J.; Lapointe, J.; Poitras, D. Schöll, E. Bright single InAsP quantum dots at telecom wavelengths in position-controlled InP nanowires: the role of the photonic waveguide. *Nano letters*, **2018**, *18*, 3047. https://doi.org/10.1021/acs.nanolett.8b00550

(26) Dalacu, D.; Mnaymneh, K.; Lapointe, J.; Wu, X.; Poole, P. J.; Bulgarini, G. Reimer, M. E. Ultraclean emission from InAsP quantum dots in defect-free wurtzite InP nanowires. *Nano letters*, **2012**, *12*, 5919. https://doi.org/10.1021/nl303327h

(27) Reimer, M. E.; Bulgarini, G.; Fognini, A.; Heeres, R. W.; Witek, B. J.; Versteegh, M. A. Dalacu, D. Overcoming power broadening of the quantum dot emission in a pure wurtzite nanowire. *Physical Review B*, **2016**, *93*, 195316. https://doi.org/10.1103/PhysRevB.93.195316

(28) Holmes, M. J.; Choi, K.; Kako, S.; Arita, M.; Arakawa, Y. Room-temperature triggered single photon emission from a III-nitride site-controlled nanowire quantum dot. *Nano letters*, **2014**, *14*, 982. https://doi.org/10.1021/nl404400d

(29) Deshpande, S.; Heo, J.; Das, A.; Bhattacharya, P. Electrically driven polarized single-photon emission from an InGaN quantum dot in a GaN nanowire. *Nature communications*, **2013**, 4, *1*. https://doi.org/10.1038/ncomms2691

(30) Deshpande, S.; Das, A.; Bhattacharya, P. Blue single photon emission up to 200 K from an InGaN quantum dot in AlGaN nanowire. *Applied physics letters*, **2013**, *102*, 161114. https://doi.org/10.1063/1.4803441

(31) Holmes, M.; Kako, S.; Choi, K.; Arita, M.; Arakawa, Y. Spectral diffusion and its influence on the emission linewidths of site-controlled GaN nanowire quantum dots. *Physical Review B*, **2015**, *92*, 115447. https://doi.org/10.1103/PhysRevB.92.115447

(32) Tatebayashi, J.; Kako, S.; Ho, J.; Ota, Y.; Iwamoto, S.; Arakawa, Y. Room-temperature lasing in a single nanowire with quantum dots. *Nature Photonics*, **2015**, *9*, 501. https://doi.org/10.1038/nphoton.2015.111

(33) Tatebayashi, J.; Ota, Y.; Ishida, S.; Nishioka, M.; Iwamoto, S.; Arakawa, Y. Highly uniform, multi-stacked InGaAs/GaAs quantum dots embedded in a GaAs

nanowire. *Applied Physics Letters*, **2014**, *105*, 103104.
https://doi.org/10.1063/1.4895597

(34) Tatebayashi, J.; Arakawa, Y. Lasing in a single nanowire with quantum dots. In *Quantum Dots and Nanostructures: Growth, Characterization, and Modeling XIV, International Society for Optics and Photonics* 10114, 1011406 (February, **2017**).
https://doi.org/10.1117/12.2256033

(35) Tatebayashi, J.; Arakawa, Y.; Recent progress in nanowire quantum-dot lasers. In *Quantum Dots and Nanostructures: Growth, Characterization, and Modeling XV, International Society for Optics and Photonics* 10543, 1054303 (**2018**, February).
https://doi.org/10.1117/12.2299672

(36) Yanase, S.; Sasakura, H.; Hara, S.; Motohisa, J. Single-photon emission from InAsP quantum dots embedded in density-controlled InP nanowires. *Japanese Journal of Applied Physics*, **2017**, *56*, 04CP04.
https://doi.org/10.7567/JJAP.56.04CP04

(37) Dorenbos, S. N.; Sasakura, H.; Van Kouwen, M. P.; Akopian, N.; Adachi, S.; Namekata, N. Fukui, T. Position controlled nanowires for infrared single photon emission. *Applied Physics Letters*, **2010**, *97*, 171106.
https://doi.org/10.1063/1.3506499

(38) Wu, J.; Ramsay, A.; Sanchez, A.; Zhang, Y.; Kim, D.; Brossard, F. Salamo, G. J. Defect-free self-catalyzed GaAs/GaAsP nanowire quantum dots grown on silicon substrate. *Nano letters*, **2016**, *16*, 504.
https://doi.org/10.1021/acs.nanolett.5b04142

(39) Bouwes Bavinck, M.; Jöns, K. D.; Zieliński, M.; Patriarche, G.; Harmand, J. C.; Akopian, N.; Zwiller, V. Photon cascade from a single crystal phase nanowire quantum dot. *Nano letters*, **2016**, *16*, 1081-1085.
https://doi.org/10.1021/acs.nanolett.5b04217

(40) Kremer, P. E.; Dada, A. C.; Kumar, P.; Ma, Y.; Kumar, S.; Clarke, E.; Gerardot, B. D. Strain-tunable quantum dot embedded in a nanowire antenna. *Physical Review B*, **2014**, *90*, 201408.
https://doi.org/10.1103/PhysRevB.90.201408

(41) Munsch, M.; Kuhlmann, A. V.; Cadeddu, D.; Gérard, J. M.; Claudon, J.; Poggio, M.; Warburton, R. J. Resonant driving of a single photon emitter embedded in a mechanical

oscillator. *Nature Communications*, **2017**, *8*, 1. https://doi.org/10.1038/s41467-017-00097-3

(42) Francaviglia, L.; Giunto, A.; Kim, W.; Romero-Gomez, P.; Vukajlovic-Plestina, J.; Friedl, M. Fontcuberta i Morral, A. Anisotropic-Strain-Induced Band Gap Engineering in Nanowire-Based Quantum Dots. *Nano letters*, **2018**, *18*, 2393. https://doi.org/10.1021/acs.nanolett.7b05402

(43) Heiss, M.; Fontana, Y.; Gustafsson, A.; Wüst, G.; Magen, C.; O'regan, D. D. Houel, J. Self-assembled quantum dots in a nanowire system for quantum photonics. *Nature materials*, **2013**, *12*, 439. https://doi.org/10.1038/nmat3557

(44) Shang, X.; Yu, Y.; Zha, G.; Li, M.; Wang, L.; Xu, J. Niu, Z. Small linewidth and short lifetime of emission from GaAs/AlAs core/shell quantum dots on the facet of GaAs nanowire. *Journal of Physics D: Applied Physics*, **2013**, *46*, 405102. https://doi.org/10.1088/0022-3727/46/40/405102

(45) Weiß, M.; Kinzel, J. B.; Schülein, F. J.; Heigl, M.; Rudolph, D.; Morkötter, S. Koblmüller, G. Dynamic acoustic control of individual optically active quantum dot-like emission centers in heterostructure nanowires. *Nano letters*, **2014**, *14*, 2256. https://doi.org/10.1021/nl4040434

(46) Yan, X.; Tang, F.; Wu, Y.; Li, B.; Zhang, X.; Ren, X. (2017). Growth of isolated InAs quantum dots on core-shell GaAs/InP nanowire sidewalls by MOCVD. *Journal of Crystal Growth*, **2017**, *468*, 185-187. https://doi.org/10.1016/j.jcrysgro.2016.11.085

(47) Yu, Y.; Zha, G. W.; Shang, X. J.; Yang, S.; Sun, B. Q.; Ni, H. Q.; Niu, Z. C. Self-assembled semiconductor quantum dots decorating the facets of GaAs nanowire for single-photon emission. *National Science Review*, **2017**, *4*, 196. https://doi.org/10.1093/nsr/nwx042
